# Assessment of facility-level antiretroviral treatment patient status utilizing a national-level laboratory cohort: Toward an understanding of system-level tracking and clinic switching in South Africa

**DOI:** 10.3389/fpubh.2022.959481

**Published:** 2022-12-15

**Authors:** Joshua P. Murphy, Khumbo Shumba, Lise Jamieson, Cornelius Nattey, Sophie Pascoe, Matthew P. Fox, Jacqui Miot, Mhairi Maskew

**Affiliations:** ^1^Health Economics and Epidemiology Research Office (HE^2^RO), Department of Internal Medicine, School of Clinical Medicine, Faculty of Health Sciences, University of the Witwatersrand, Johannesburg, South Africa; ^2^Department of Global Health, School of Public Health, Boston University, Boston, MA, United States

**Keywords:** HIV, retention in care, clinic switching, mobility, silent transfers, misclassification

## Abstract

**Background:**

Most estimates of HIV retention are derived at the clinic level through antiretroviral (ART) patient management systems, which capture ART clinic visit data, yet these cannot account for silent transfers across HIV treatment sites. Patient laboratory monitoring visits may also be observed in routinely collected laboratory data, which include ART monitoring tests such as CD4 count and HIV viral load, key to our work here.

**Methods:**

In this analysis, we utilized the NHLS National HIV Cohort (a system-wide viewpoint) to investigate the accuracy of facility-level estimates of retention in care for adult patients accessing care (defined using clinic visit data on patients under ART recorded in an electronic patient management system) at Themba Lethu Clinic (TLC). Furthermore, we describe patterns of facility switching among all patients and those patients classified as lost to follow-up (LTFU) at the facility level.

**Results:**

Of the 43,538 unique patients in the TLC dataset, we included 20,093 of 25,514 possible patient records (78.8%) in our analysis that were linked with the NHLS National Cohort, and we restricted the analytic sample to patients initiating ART between 1 January 2007 and 31 December 2017. Most (60%) patients were female, and the median age (IQR) at ART initiation was 37 (31–45) years. We found the laboratory records augmented retention estimates by a median of 860 additional active records (about 8% of all median active records across all years) from the facility viewpoint; this augmentation was more noticeable from the system-wide viewpoint, which added evidence of activity of about one-third of total active records in 2017. In 2017, we found 7.0% misclassification at the facility-level viewpoint, a gap which is potentially solvable through data integration/triangulation. We observed 1,134/20,093 (5.6%) silent transfers; these were noticeably more female and younger than the entire dataset. We also report the most common locations for clinic switching at a provincial level.

**Discussion:**

Integration of multiple data sources has the potential to reduce the misclassification of patients as being lost to care and help understand situations where clinic switching is common. This may help in prioritizing interventions that would assist patients moving between clinics and hopefully contribute to services that normalize formal transfers and fewer silent transfers.

## Introduction

Continuity in HIV care from antiretroviral treatment (ART) initiation to virologic suppression is essential for the overall health of HIV-infected patients and the prevention of HIV transmission at the population and individual levels (1, 2). Most estimates of HIV retention are derived at the clinic level through HIV patient management systems, which capture ART clinic visit data, yet these cannot account for silent transfers across sites (3). Despite evidence that South African patients on ART are highly mobile (4, 5) and that “mobility continues to place individuals at risk of HIV acquisition as well as onward transmission” (6), there are few estimates quantifying silent transfers and clinic switching (7–9).

However, patient laboratory monitoring visits may also be observed in routinely collected laboratory data, which include results of ART monitoring tests such as CD4 count and HIV viral load. We previously utilized laboratory test results from South African National Health Laboratory Service (NHLS) to create the NHLS National HIV Cohort—a longitudinal cohort of patients accessing HIV care in South Africa's public sector between 2004 and 2018 (10, 11). In this analysis, we utilized the NHLS National HIV Cohort data (a system-wide viewpoint) to investigate the accuracy of facility-level estimates of retention in care (defined using clinic visit data on patients under ART recorded in an electronic patient management system). Furthermore, we described patterns of facility switching among all patients and those classified as lost to follow-up (LTFU).

## Methods

### Data sources

We utilized two sets of routinely collected public sector data, which are as follows:

1) Right to Care Clinical Cohort: facility-level data
Right to Care South Africa (RTC) is a South African NGO that has engaged in South African HIV program since the early 2000s. Through the electronic medical record (EMR) database TherapyEdge™ (TE), RTC has collected patient-level data on clinical visits, treatment, and laboratory monitoring of patients. These data have been developed into the RTC Clinical Cohort representing >100,000 patients at multiple facilities across Gauteng, Limpopo, and Mpumalanga (12). In this study, we analyzed facility-level electronic medical records from a cohort of patients accessing care at one of South Africa's longest standing HIV treatment clinics, the Themba Lethu Clinic (TLC) in Gauteng Province, which was established in 2004 and has a detailed profile (12).
2) The NHLS National HIV Cohort: national-level laboratory data
The NHLS conducts all routine laboratory monitoring for the public sector HIV program in South Africa and maintains these electronic records. As these data lack a unique patient identifier, a linkage algorithm was developed to identify unique patients (and their associated laboratory results) through the demographic data available using probabilistic and network-based linkage methodology, creating the NHLS National HIV Cohort (10). This cohort is a national-level longitudinal record of all HIV-associated laboratory tests conducted at public sector facilities in South Africa. As such, the cohort can observe a system-wide viewpoint of patients accessing HIV care (using laboratory testing records as a proxy) across all facilities nationally. Data for the cohort are currently available from 2004 to 2018 for all provinces with an exception of KwaZulu-Natal, where data are observed from 2009.

### Study population and data inclusion

In our analysis, we included records for adult patients aged >18 years with a documented ART start date at TLC during the study period of 2007–2017. Visit data were not available for the full year of 2018, so we limited the data to those who started ART before the end of 2017 to allow for a sufficient follow-up to monitor LTFU. Records for these patients were then linked to the NHLS National HIV Cohort using both deterministic and probabilistic matching approaches (10). Since laboratory results exist in both the RTC Clinical Cohort and the NHLS National HIV Cohort data, we used the laboratory test type, test date, and numeric test result as our linking variables (10).

### Study variables

The primary outcome of this study was the misclassification of patient retention in HIV care. We defined patient retention from a system-wide perspective as well as at the facility level (shown in Table 1). Specifically, we defined facility-level retention in four ways as follows:

A) Visit record retention: Patients were considered active in care during each calendar year under study if a clinic visit at TLC was observed in the electronic medical record (EMR) of the site during the corresponding period.
B) Laboratory record retention: A patient was considered active in care during each calendar year under study if a laboratory test was observed in the NHLS National HIV Cohort records for the originating facility during that corresponding period.
C) Proxy gold standard of patient activity at TLC: An estimated total number of patients active in care by combining patients classified by either the facility-level visit record (A) or facility-level laboratory record (B) for each calendar year. This observed total number of patients was deduplicated to give a denominator of total unique patients active in care each year at TLC.
D) Patient record retention: The classification of the patient status was recorded in the EMR at the facility (active in care, lost to follow-up, transferred out, and deceased). Patient record outcomes (active in care, lost to follow-up, transferred out, and deceased) were not included as part of this gold standard as these outcomes are manually entered, rather than directly observed data.

**Table 1:** Description of study outcome variables.

**Attributes**	**Facility-level retention viewpoint**				**System-wide retention viewpoint**
	**A Visit record retention**	**B Laboratory record retention**	**C Gold standard proxy of patient activity at TLC**	**D Patient record retention**	**E Health-system retention/proxy gold-standard of any activity**
Patients active in care if:	A clinic visit recorded	A laboratory test recorded	Either clinic visit or lab test recorded	Patient classified as active in care on medical record	A clinic visit or laboratory test was observed
Where:	Recorded at TLC	Taken at TLC	Either visit record or lab record	At the facility where they initiated ART	At any facility in the NHLS cohort including TLC
When:	During each calendar year	During each calendar year	During each calendar year	Assessed within each calendar year or latest outcome data	During each calendar year
Data source:	Facility EMR	NHLS national HIV cohort	Facility EMR or NHLS HIV cohort	Facility EMR	NHLS national HIV cohort or from sources described in Column A at TLC

EMR, electronic medical record; TLC, Themba Lethu Clinic.

However, in our definition of system-wide retention, a patient was considered retained if the patient was active in care by having a patient visit or laboratory test observed at any facility included in the NHLS National HIV Cohort data during the corresponding calendar year (column E).

Our secondary outcome was clinic switching, which was defined by examining all records as well as records indicating a silent transfer. We defined a silent transfer to have occurred if a patient had a lost to follow-up (LTFU) outcome recorded for at least 6 months in the electronic patient record at TLC, and evidence of a laboratory test after the LTFU outcome date observed in any other facility besides TLC within the NHLS National HIV Cohort.

### Data analysis

We described the study population characteristics at ART initiation using descriptive statistics including proportions for categorical variables and medians with their corresponding interquartile ranges (IQRs) for continuous variables. Our analysis focused on reporting two main measures: (1) the proportion of people classified as active in care in the entire dataset and (2) the proportion of records accurately classified as active in care compared with the gold standard definition by either the visit record or the laboratory record. To do this, we report on the proportion of patients correctly classified as active in care by each retention definition both at the facility and system-wide levels as compared to the proxy gold standard.

We quantified misclassification for the single year of 2017 in two ways: misclassification at the facility level and misclassification at the system-wide level. We considered a patient misclassified at the facility level if either the (1) visit record or (2) the laboratory record did not concur with the facility-level gold standard retention classification. We defined system-wide misclassification to have occurred if either (1) a system-wide laboratory record or (2) a facility-level visit record did not agree with the system-level gold standard retention classification.

Lastly, we described observed clinic switching as the proportion of patients with an observed laboratory record at any facility within the NHLS cohort other than TLC (observed clinic switch), and among those classified as LTFU at TLC each year between 2007 and 2017. Clinic switching (presumed mobility) was summarized at the facility and provincial levels using frequencies and simple proportions.

### Ethical considerations

Ethical approval for this analysis was granted by the Human Research Ethics (Medical) Committee of the University of the Witwatersrand Human Research Ethics Committee (Medical) M190981 and M1902105. Data were anonymized, and access was limited to the study team; our work also followed the Strengthening the Reporting of Observational Studies in Epidemiology (STROBE) guidelines (Supplementary material 1) (13).

## Results

### Study population

**Figure 1:**
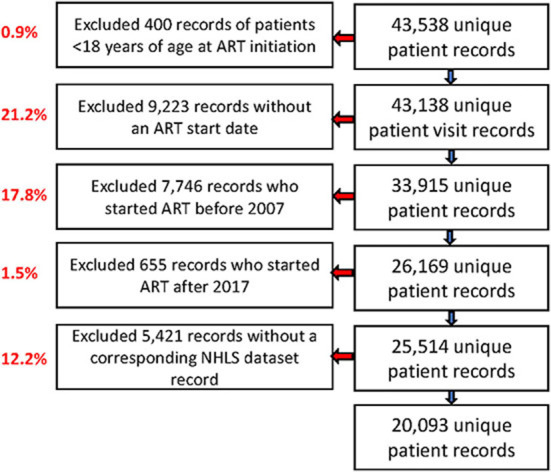
Study flow chart showing the selection process of ART patients records from Themba Lethu Clinic (TLC) and National Health Laboratory Service (NHLS) laboratory record data from the NHLS National HIV Cohort.

Of the 43,538 unique patient records in the TLC dataset, we excluded 400 records (0.9%) of individuals younger than 18 years, 9,223 records (21.2%) without an ART start date, 7,746 records (17.8%) with ART start dates before 2007, 655 records (1.5%) with ART start dates after 2017, and 5,421 records (12.5%) that were not observed in the NHLS National HIV Cohort (Figure 1). In our analysis, we included 20,093 of 25,514 remaining patient records (78.8%), and we successfully linked those with the NHLS National Cohort. Most (60%) of these patients were female, with a median age at ART initiation (IQR) of 37 years (31–45; Table 2). More than half of these patients (68.5%) initiated on ART had a baseline CD4 count of < 200 cells/mm^3^, with a median CD4 count at initiation of 136 cells/mm^3^ (52–230). Over 70% of the cohort-initiated treatment during the period 2007–2012, with a median time on ART (IQR) of 6.7 (4.3–8.6) years at the database censor date of 31 December 2017.

**Table 2:** Characteristics at ART initiation of patients forming the study sample from Themba Lethu Clinic in Johannesburg, South Africa (*n* = 20,093).

**Variable**	***n* (%)/median (IQR)**
Total *N*	20,093
**Gender**
Female	12,141 (60.4%)
Male	7,952 (39.6%)
**Age at ART initiation (years)**
Median (IQR)	37.3 (31.2–44.8)
18–29	4,107 (20.4%)
30–34	3,987 (19.8%)
35–39	3,913 (19.5%)
40–44	3,171 (15.8%)
>45	4,915 (24.5%)
**ART start dates by year (grouped)**
2007–2008	4,038 (20.1%)
2009–2010	5,121 (25.5%)
2011–2012	4,970 (24.7%)
2013–2014	2,496 (12.4%)
2015–2017 (3 years)	3,468 (17.3%)
**Duration on ART (years)**
Median years (IQR)	6.7 (4.3–8.6)
< 1	1,001 (5.0%)
1–3	2,467 (12.3%)
>3–7	7,466 (37.2%)
>7	9,159 (45.6%)
**Baseline CD4 category (cells/μl)**
Median (IQR)	136 (52–230)
< 50	4,106 (24.7%)
51–100	2,547 (15.3%)
100–200	4,746 (28.5%)
201–350	3,496 (21.1%)
≥350 and up	1,728 (10.4%)
Missing (excluded from %)	3,470

ART: antiretroviral therapy; CD4, CD4 count; IQR: interquartile range.

### Estimates of retention in care

By the end of the study period in 2017, 13,981 (70%) of the total study sample were considered active in care at the system-wide level. Estimates of correctly classifying patients as active in care increased over the study period across all definitions of retention (Table 3). The proportion correctly classified as active by laboratory records was consistently above 92% both at the facility and system-wide levels.

**Table 3:** Facility-level and system-wide retention estimate of Themba Lethu Clinic (TLC) 2007–2017.

**Year**	**Facility-level retention definitions**				**System-wide viewpoint definition**			
	Total active in care at TLC*	**Visit record retention *n* (%)**	**Laboratory record retention *n* (%)**	**Patient record retention *n* (%)**	Total active in care across all facilities*	**Visit record retention *n* (%)**	**Laboratory record retention *n* (%)**	**Patient record retention *n* (%)**
2007	3,236	2,301 (71.1%)	3,125 (96.6%)	1,773 (54.8%)	4,839	2,301 (47.6%)	4,757 (98.3%)	1,773 (36.6%)
2008	4,838	3,770 (77.9%)	4,631 (95.7%)	3,211 (66.4%)	6,651	3,770 (56.7%)	6,488 (97.5%)	3,211 (48.3%)
2009	6,701	5,632 (84.0%)	6,387 (95.3%)	4,889 (73.0%)	8,498	5,632 (66.3%)	8,279 (97.4%)	4,889 (57.5%)
2010	8,572	7,454 (87.0%)	8,160 (95.2%)	6,508 (75.9%)	10,572	7,454 (70.5%)	10,337 (97.8%)	6,508 (61.6%)
2011	9,956	8,975 (90.1%)	9,304 (93.5%)	8,072 (81.1%)	11,903	8,975 (75.4%)	11,513 (96.7%)	8,072 (67.8%)
2012	10,531	9,671 (91.8%)	9,980 (94.8%)	9,019 (85.6%)	12,572	9,671 (76.9%)	12,112 (96.3%)	9,019 (71.7%)
2013	10,440	9,591 (91.9%)	9,714 (93.0%)	8,974 (86.0%)	12,743	9,591 (75.3%)	12,218 (95.9%)	8,974 (70.4%)
2014	10,471	9,628 (91.9%)	9,688 (92.5%)	9,357 (89.4%)	13,096	9,628 (73.5%)	12,424 (94.9%)	9,357 (71.4%)
2015	10,812	10,055 (93.0%)	10,353 (95.8%)	9,697 (89.7%)	13,525	10,055 (74.3%)	13,163 (97.3%)	9,697 (71.7%)
2016	11,097	10,518 (94.8%)	10,530 (94.9%)	9,785 (88.2%)	13,939	10,518 (75.5%)	13,556 (97.3%)	9,785 (70.2%)
2017	10,802	10,481 (97.0%)	10,367 (96.0%)	9,308 (86.2%)	13,981	10,481 (75.0%)	13,683 (97.9%)	9,308 (66.6%)

*Visit records + NHLS lab visit records.

From the facility-level viewpoint, laboratory record estimates of retention classified the largest proportion of active patients correctly, outperforming visit record retention in nearly every year (reporting higher retention). Laboratory records added a median of 860 (about 8% of all median active records across all years) additional correctly classified active records (range from 321 in 2017 to 1,118 in 2010) to retention estimates (Supplementary material 2). The proportion of patients correctly classified as active in care by visit records increased from 71.1% in 2007 to 97.0% in 2017. From the period of 2007 to 2017, the visit record retention misclassified 6.6% more records on average compared with laboratory data retention estimates, while patient record data misclassified 15.2% more records than laboratory record retention estimates (Supplementary material 2). By 2017, visit record data outperformed the laboratory data, although both were still noticeably higher than the patient record retention status estimates.

**Figure 2:**
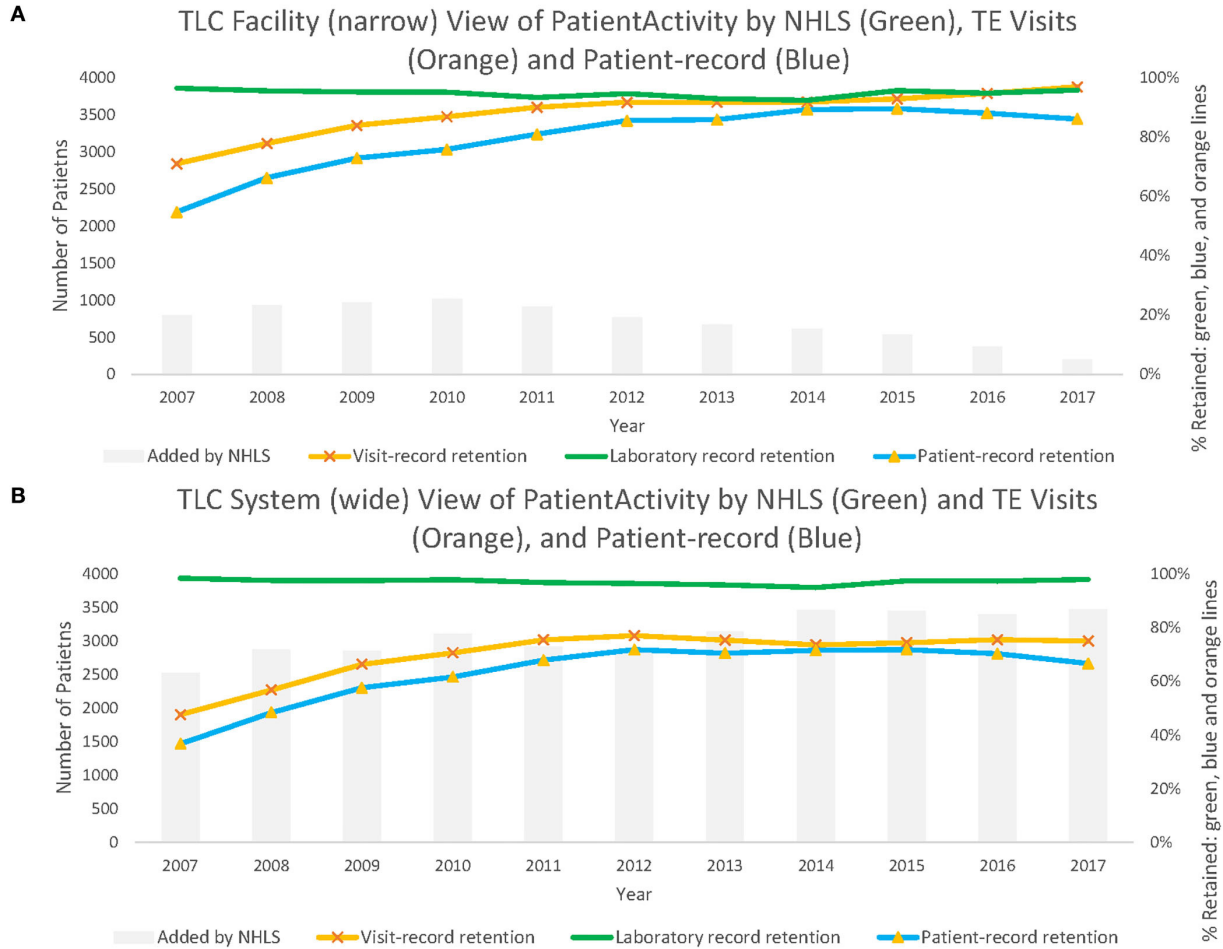
Panel **(A)** shows patient active in care at Themba Lethu Clinic (TLC) from the facility-level viewpoint. While Panel **(B)** shows patient active in care across the system-wide viewpoint from those records originating from TLC.

From the system-wide retention viewpoint, visit record data correctly classified a maximum of 76.9% of patients who were identified as active in care at the health system level (Table 3). Patient record estimates of retention were the least accurate in classifying retention correctly from the system-wide viewpoint. This is in contrast to the facility-level estimates, which increased steadily from 54.8% in 2007 to 86.2% in 2017. The potential augmentation from laboratory records was larger than the visit record data and patient record data throughout. During the reported time frame, the visit record data were, on average, 27.3% lower than the laboratory record data, and the patient record data were 33.9% lower than the laboratory record data (Supplementary material 2). In 2017, we observed increases of more than 20% between both measures to the laboratory record retention estimate, classifying patients as active for 97.9% of records. Not included in the table but visible in Figure 2, this augments retention by adding a median of 2,538 patient records (making up 41.8% of the estimated total active patients in 2007) and 3,500 (33.4% of the total estimated) additional records in 2017.

Over time, facility-level laboratory estimates appear to largely outperform the visit record data and patient record retention estimates for each year until 2012 (Figure 2A). While in Figure 2B, we note that system-wide laboratory data-based estimates of retention consistently correctly classify retention estimates at higher proportions than visit record or patient record data throughout.

Of 10,802 patients reported as retained in 2017 from the facility-level viewpoint, we observed 756 (7.0%) records with misclassification errors (Table 4); of these, 435 (4.0%) had a visit record and no laboratory record, while 321 (3.0%) had a laboratory record and no visit record (not shown). From the system-level viewpoint, of the 13,981 observed active records in 2017, we found 3,798 (27.2%) records with a misclassification error; of these, 298 (2.1%) records had a visit record and no laboratory record, while 3,500 (25.0%) had a laboratory record and no visit record. We observed that misclassified records at the system-level viewpoint were: more often female (65.6% female patients vs. 60.9% male patients), younger in years of age at ART initiation (median age 36.3 vs. 38.0 years), on ART for fewer years (median years on ART 2.18 vs. 5.6), and at a lower baseline CD4 (cells/mm^3^ 144 vs. 157).

**Table 4:** Facility-level and system-wide viewpoints of correctly classified and misclassified records (2017).

	**Facility-level viewpoint**		**System-level viewpoint**	
Total active		10,802		13,981
**Variable**	**Misclassified**	**Correctly classified**	**Misclassified**	**Correctly classified**
	756 (7.0%)	10,046 (93.0%)	3,798 (27.2%)	10,183 (72.8%)
**Gender**	
Female	444 (58.7%)	6,112 (60.8%)	2,492 (65.6%)	6,204 (60.9%)
Male	312 (41.3%)	3,934 (39.2%)	1,306 (34.4%)	3,979 (39.0%)
Age at ART initiation	
Median (IQR)	37.8 (31.4–44.8)	38.0 (31.7–45.2)	36.3 (30.3–43.9)	38.0 (31.7–45.3)
**Time on ART (years)**	
Median (IQR)	3.5 (1.5–6.1)	5.6 (2.6–7.6)	2.2 (1.0–4.1)	5.6 (2.6–7.6)
< 1	135 (17.9%)	1,085 (10.8%)	965 (25.4%)	1,101 (10.8%)
1–3	209 (27.6%)	1,804 (18.0%)	1,396 (36.8%)	1,833 (18.0%)
>3 to 7	281 (37.2%)	3,954 (39.4%)	1,207 (31.8%)	4,021 (39.5%)
>7	131 (17.3%)	3,203 (31.9%)	230 (6.1%)	3,228 (31.7%)
**Baseline CD4 category**	
Median (IQR)	150 (62–260)	157 (62–263)	144 (62–226)	157 (62–263)
< 50 (0)	131 (21.2%)	1,772 (21.5%)	660 (21.4%)	1,796 (21.5%)
51–100 (1)	96 (15.5%)	1,121 (13.6%)	500 (16.2%)	1,137 (13.6%)
100–200 (2)	165 (26.7%)	2,226 (27.0%)	965 (31.3%)	2,255 (27.0%)
201–350 (3)	140 (22.6%)	1,969 (23.9%)	670 (21.8%)	1,998 (23.9%)
>350 and up (4)	87 (14.0%)	1,144 (13.9%)	284 (9.2%)	1,162 (13.9%)
Missing (excluded from %)	137	1,814	719	1,835

ART, antiretroviral therapy; CD4, CD4 count; IQR, interquartile range.

### Description of patient mobility

Of the total study population (*n* = 20,093), we observed 5,414 (27%) patients with laboratory records at TLC only, while the remaining 14,679 (73%) records had evidence of laboratory monitoring from at least one facility other than TLC (Table 5). There were five patterns of facility movement observed: no clinic switching (laboratory records only observed at TLC; *n* = 5,414), clinic switch from another clinic to TLC (*n* = 2,089; 10.4%; laboratory records observed at other facilities prior to records at TLC), clinic switch from TLC to another facility (*n* = 3,555; 17.7%; laboratory records observed at other facilities after records at TLC), and dual clinic access (*n* = 5,568; 27.7%) (laboratory records observed at another clinic while accessing care at TLC in the same year, and multiple clinic switches crossing over between TLC and at least one other facility across multiple years (*n* = 3,467; 17.3%).

**Table 5:** Types of facility movement among Themba Lethu Clinic (TLC) patients (*n* = 20,093).

**Variable**	**Total**
*N*	20,093
**Patient mobility pattern**
No clinic switching	5,414 (26.9%)
Clinic switch prior to TLC	2,089 (10.4%)
Clinic switch after TLC	3,555 (17.7%)
Dual clinic access (TLC and another facility during the same year)	5,568 (27.7%)
Multiple clinic switches (crossover*)	3,467 (17.3%)

*Multiple clinic switches crossing over between Themba Lethu Clinic and at least one other facility across multiple years.

We describe two groups of patients with evidence of possible clinic switching: (1) patients with observed clinic switching of the entire dataset 14,679/20,093 (73.1%) and (2) likely “silent transfers” from the dataset (Table 6). The silent transfers were based on those with an LTFU outcome date that fell before a laboratory record at a facility other than TLC in 2017, and we observed 1,134 of 20,093 (5.6%) of such records. The groups were similar, but notably, we observed differences between the entire dataset and both the silent transfer records and all records with evidence of clinic switching. For example, the entire dataset was 60.4% female while both the silent transfers and all patients with evidence of clinic switching were more female, 62.2% (*n* = 705) of silent transfer records and 64.4% (*n* = 9,446) of all patient records with evidence of clinic switching were female. We found that silent transfers were also on ART for a shorter amount of time than all records with clinic switching as well as the entire dataset, with median years on ART (IQR) of 1.8 (0.7–3.3) for silent transfers, 3.1 (1.1–6.1) for all records with evidence of clinic switching, and 6.7 (4.3–8.6) for the entire dataset. The age at ART initiation was 34.4 (29.0–40.7) years for the silent transfer group, which was younger than that for all records with clinic switching, 36.8 (30.8–44.3), and the entire dataset, 37.3 (31.2–44.8). We also observed records with laboratory results from as many as 12 facilities in the dataset (from all groups), and the median laboratory records were 2 (IQR: 1–3) facilities. Of all records with evidence of movement, 22.9% (*n* = 3,368) had a switch outside of Gauteng compared with 31.0% (*n* = 351) of the silent transfers. The most common provinces for movement were KwaZulu-Natal (7.7% of silent transfers vs. 4.4% of all records with movement), North West (6.6 vs. 5.4%), Eastern Cape (5.6 vs. 3.3%), and Limpopo (4.8 vs. 3.9%). About 75% of the silent transfers had their LTFU outcomes before 2015, but about a quarter had their LTFU outcome between 2015 and 2017.

**Table 6:** Clinic switching and silent transfers among patients with HIV accessing care at Themba Lethu Clinic (TLC).

**Variable**	**Observed silent transfers**	**Evidence of clinic switching**
*N*	1,134	14,679
Gender
Female	705 (62.2%)	9,446 (64.4%)
Male	429 (37.8 %)	5,233 (35.7%)
**Age at ART initiation**
Median (IQR)	34.40 (29.0–40.7)	36.8 (30.8–44.3)
**Time on ART (years)**
Median (IQR)	1.8 (0.7–3.3)	3.1 (1.1–6.1)
**Provincial and inter-provincial movement**
Clinic switch within Gauteng	902 (79.5%)	13,648 (93.0%)
Clinic switch outside Gauteng	351 (31.0%)	3,368 (22.9%)
Clinic switched both within and outside Gauteng	119 (10.5%)	2,337 (15.9%)
Median number (IQR) of facilities records included (range)	2 (1–3) (1–12)	2 (1–3) (1–12)
**Records with evidence of presence in the following provinces (IQR) (range):**
Eastern Cape	63 (5.6%)	490 (3.3%)
Free State	29 (2.6%)	324 (2.2%)
KwaZulu-Natal	87 (7.7%)	650 (4.4%)
Limpopo	54 (4.8%)	574 (3.9%)
Mpumalanga	28 (2.5%)	329 (2.2%)
Northern Cape	11 (1.0%)	104 (0.7%)
North West	75 (6.6%)	785 (5.4%)
Western Cape	19 (1.7%)	263 (1.8%)
**Evidence of activity in number of provinces:**
1	329 (29.0%)	3,017 (20.6%)
2	17 (1.5%)	215 (1.5%)
3	1 (0.1%)	24 (0.2%)

## Discussion

We described the accuracy of record classification of 20,093 ART patient records at Themba Lethu Clinic (TLC) from 2007 to 2017 from three viewpoints: (1) visit record retention, (2) laboratory record retention, and (3) patient record retention. The second viewpoint derived from the NHLS National HIV Cohort (10), provided a valuable system-level view that served as our proxy “gold standard” of activity and allowed an exploration of patient mobility and clinic switching–in and out of Themba Lethu Clinic. Across the time frame, we noted a high agreement in the classification of records, particularly during the last year of the study period in 2017. Across all years, the lowest agreement was from the patient record outcomes (active in care, lost to follow-up, transferred out, and deceased).

We highlight four main findings: (1) The laboratory records augmented retention estimates by a median of 860 additional active records (about 8% of all median active records across all years) from the facility viewpoint, and this augmentation was more noticeable from the system-wide viewpoint, which added evidence of activity of about one-third of total active records in 2017; (2) in 2017, we found 7.0% misclassification at the facility-level viewpoint, a gap which is potentially solvable through data integration/triangulation; (3) across our records, we observed 1,134 of 20,093 (5.6%) of the total records were silent transfers, which had noticeably more female and younger patients than the entire records; and (4) of all records with evidence of movement, 3,368 (22.9%) had a switch outside of Gauteng, while common clinic switching included the following provinces: KwaZulu-Natal (7.7% of silent transfers vs. 4.4% of all records with movement), North West (6.6 vs. 5.4%), Eastern Cape (5.6 vs. 3.3%), and Limpopo (4.8 vs. 3.9%).

### Importance of findings

National-level patient records, such as laboratory cohorts, have the potential to improve facility-based estimates of patient care status. Our findings support others who have chronicled the importance and possibility of data integration theoretically (14) and practically (15). The National Department of Health has undertaken a deduplication process at the facility level, ongoing since early 2020, to provide a “platform to assist healthcare practitioners to improve patient management and data management processes across healthcare facilities.” However, this approach is limited in that “the patient clinical record is the only source document that can be used” for verification (16). The platform used for deduplication is TIER.Net (a non-networked electronic register), a bridging solution toward a networked electronic medical record that has yet to be achieved (3). In the Western Cape, Boulle et al. (15) have documented that the person-level integration from multiple sources is possible while maintaining the security of patient's personal information. In preparation for the National Health Insurance, a South African planned universal healthcare coverage plan, Katurura and Cilliers (17) reported that the South African National Department of Health (NDoH) needs to be able to understand patient movements as patients move between healthcare facilities. We have provided more information toward that understanding of movement and the gaps/magnitude to measuring optimal patient retention.

### Recent literature and the “revolving door” of engagement with HIV care

**Figure 3:**
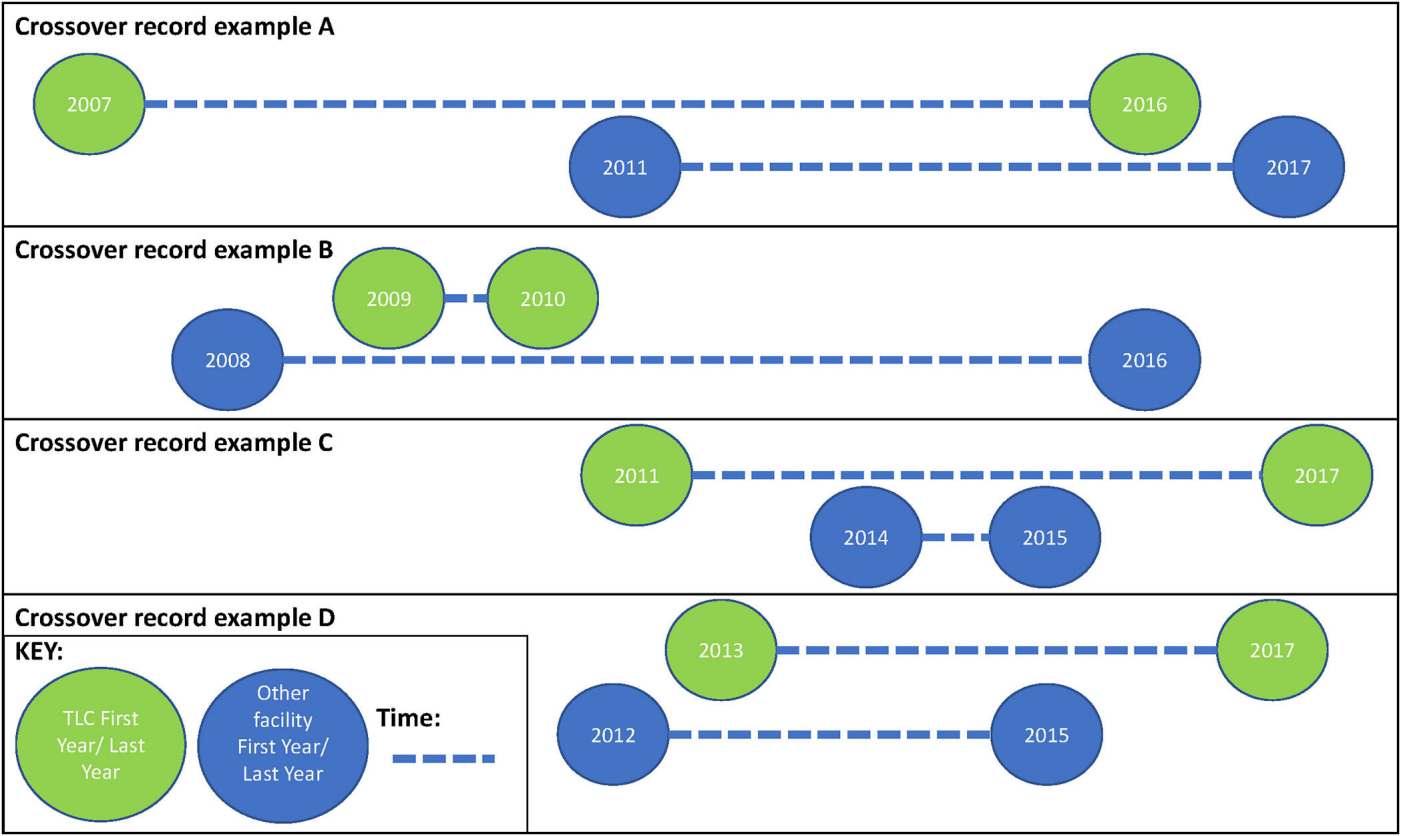
Illustrative scenarios of multiple clinic switches “cross-overs”.

We have highlighted that South African patients undergoing ART are highly mobile (4, 5), and that this mobility may affect health outcomes as well as onward HIV transmission (7–9). We also noted that there are limited estimates of the magnitude of silent transfers, while clinic switching is not well documented in South Africa (7–9). However, estimates that do exist range between 2.5% and 36.7% (7, 8), and our estimate of 28.6% falls within this range. Our findings support the implications that the patient care journey should be viewed in a cyclical nature. Figure 3 shows the scenarios that we observed in the data (which are presented in Table 4 in the row “multiple clinic switches,” representing 17% of the dataset). All this proves patient movement between facilities is complex.

Our findings align with Kaplan (2017) and others who have called for “interlinkage” of routine health information systems (5, 7, 14). Whether the focus should be to reduce disengagement (7) and/or speed up the process at the point they re-engage changes the paradigm in which the HIV continuity of care is conceived away from a “revolving door” (18), or modernize the health information system (17). Political will is required, as evidenced in the Western Cape's provincial data center (15).

Bisnauth et al. (19) have reported the implementation of the “Welcome Back” campaign developed by Médecins Sans Frontiers and designed to normalize, support, and empower clients returning to care, including after a clinic switch. One of their key findings suggests there is still progress to be made in normalizing return to care as a quarter of healthcare workers reported that patients are sent to the back of the queue and that transfer letters are required to receive care (19). Similarly, Rees et al. (20) have called for being supportive of patients who are re-initiating ART after treatment interruptions, suggesting a need to remove judgment of patients who may be worried about re-initiation.

An examination of the latest available migration dynamics report from StatsSA triangulates with our descriptions of patient movement. Of the seven major inter-provincial migration corridors, our findings were in concordance with the six that included Gauteng (21). The only difference was that StatsSA noted top four inter-provincial migrations from Gauteng to the following provinces in the order as follows: (1) Limpopo, (2) KwaZulu-Natal, (3) Eastern Cape, and (4) North West, while we observed (of all records with observed movement) the following order: (1) North West, (2) KwaZulu-Natal, (3) Limpopo, and (4) Eastern Cape (21). Lastly, our observed silent transfers had KwaZulu-Natal at the top position. This triangulation of data sources also emphasizes the importance of including migration/mobility as a public health research priority (22).

**Figure 4:**
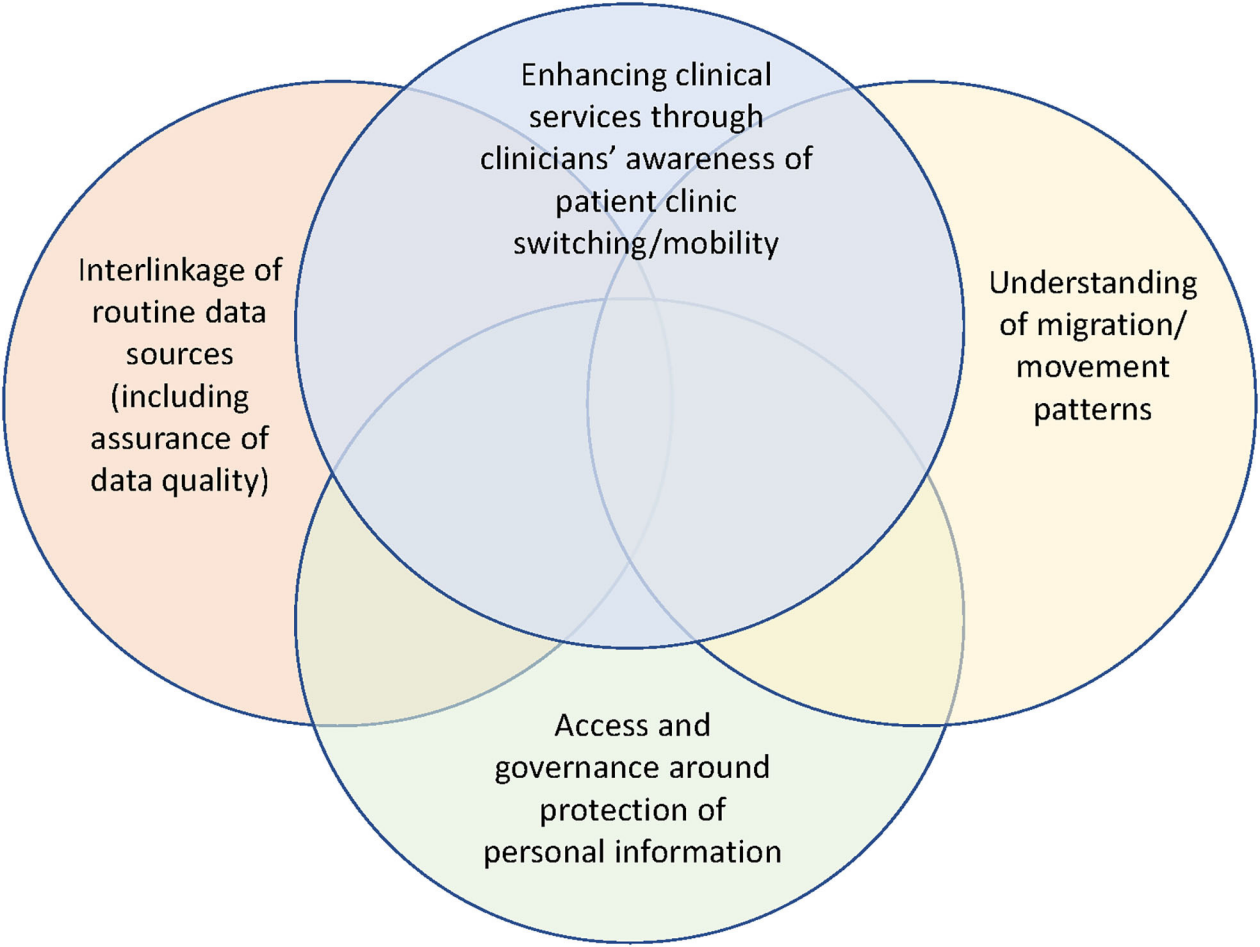
Integration of potential research areas for further exploration regarding patient outcomes misclassification, understanding patient movement and understanding ART retention.

Lastly, our findings also align in some respect with misclassification research by others in South Africa, notably to the study by Etoori et al. (23), who shared the consequences of misclassifications of outcomes such as inaccuracies in forecasting and over-reporting of patient LTFU outcomes. Figure 4 shows a Venn diagram that illustrates potential research areas for further exploration of patient outcomes misclassification, patient movement, and understanding ART retention.

### Strengths and limitations

Our primary strength was our access to relatively high-quality historical patient-based data (12) and the NHLS HIV Cohort (10). We also explored the 11-year time frame that covered large growth in both Johannesburg and South Africa's HIV treatment program, a program that has experienced several policy changes, two of which includes universal treatment for everyone (2016) and same-day ART initiation (2017) that fell within our time frame (24, 25). Our findings also contribute to the growing literature on the importance of enhancing the accuracy of medical records (26). Lastly, these findings also highlight how patients on ART have very different paths toward care and as such differentiated care that tailors to the needs of different population groups is an important consideration for optimizing adherence and retention (4, 27).

Our primary limitation is that these findings lacked verification from paper-based records. Second, the nature of our analysis did not allow for measuring switches that happened within the same year, except for the identification of silent transfers where precise dates were known and could be accounted for. Similarly, we counted the number of facilities at which a patient had a laboratory record within the same year; however, we could not count the total number of switches. Of note, TLC has integrated laboratory records into their TherapyEdge database since 2012 and is therefore not representative of typical public sector service points. Despite this, it is possible that there were still instances of misclassification in our analysis, which could have been due to laboratory results that occurred at the transition from 1 year to the next and thus were not classified in the correct year alongside the clinical visits. Lastly, we did not have data to assess movement during the COVID-19 pandemic, which is of critical importance (28, 29).

## Conclusion

We document the potential positive augmentation of using a National HIV Cohort (based on NHLS laboratory data) to create potentially more accurate estimates of retention. Integration of multiple data sources has the potential to reduce misclassification of patients as being lost to care and help to understand situations where clinic switching is common. This would help in prioritizing interventions that would assist patients moving between clinics and hopefully contribute to services that normalize formal transfers and fewer silent transfers.

## Data availability statement

The data analyzed in this study is subject to the following licenses/restrictions. Information on the datasets analyzed for this study can be found at: https://pubmed.ncbi.nlm.nih.gov/22434860/ and https://www.biorxiv.org/content/10.1101/450304v1. South Africa's Protection of Personal Information as well as conditions of the University of the Witwatersrand Human Research Ethics Committee and HE^2^RO's internal data handling policies will limit access to underlying data. Data accessibility is documented in this paper: Bor et al. (10) and Fox et al. (12). Requests to access these datasets should be directed at: information@heroza.org.

## Ethics statement

Ethical approval for this analysis was granted from the Human Research Ethics (Medical) Committee of the University of the Witwatersrand Human Research Ethics Committee (Medical) M190981 and M1902105. Data were anonymized and access was limited to the study team; our work also followed the Strengthening the Reporting of Observational Studies in Epidemiology (STROBE) guidelines.

## Author contributions

The study was conceived by JPM and MM. Data curation was conducted by JPM, KS, and CN. Analyses were conducted by JPM with input from KS, LJ, CN, and MM. The manuscript was drafted by JPM with input from KS, LJ, SP, MF, JM, and MM. All authors contributed to the interpretation of the findings and read and approved the final manuscript.
